# Mining the Urban Sprawl Pattern: A Case Study on Sunan, China

**DOI:** 10.3390/s8106371

**Published:** 2008-10-14

**Authors:** Ronghua Ma, Chaolin Gu, Yingxia Pu, Xiaodong Ma

**Affiliations:** 1 Nanjing Institute of Geography and Limnology, Chinese Academy of Sciences, Nanjing, PO Box 210008, P.R. China; E-mail: mrhua2002@gmail.com (R. M.); 2 School of Architecture, Tsinghua University, Beijing, PO Box 100084, P.R. China; 3 College of Geography & Marine, Nanjing University, Nanjing, PO Box 2100913, P.R. China; 4 College of Urban and Environmental Science, Xuzhou Normal University, Xuzhou, PO Box 221009, P.R. China

**Keywords:** Urban sprawl pattern, Spatial autocorrelation, Fractal dimension, Sunan, China

## Abstract

China's urbanization is going into a fast development stage. This paper focuses on the recent evolution of an urbanized area – Sunan, the southern part of Jiangsu province in the Yangtze River Delta in China – by means of complementary approaches, especially different fractal and autocorrelation measures. The research shows that Sunan's urban clusters are becoming more and more homogenous and compact and are growing up along the important transportation axes. The enriching discussion of the findings establishes the links between the morphology of urban sprawl and recent socio-economic changes in China.

## 1. Introduction

**Figure 1.**
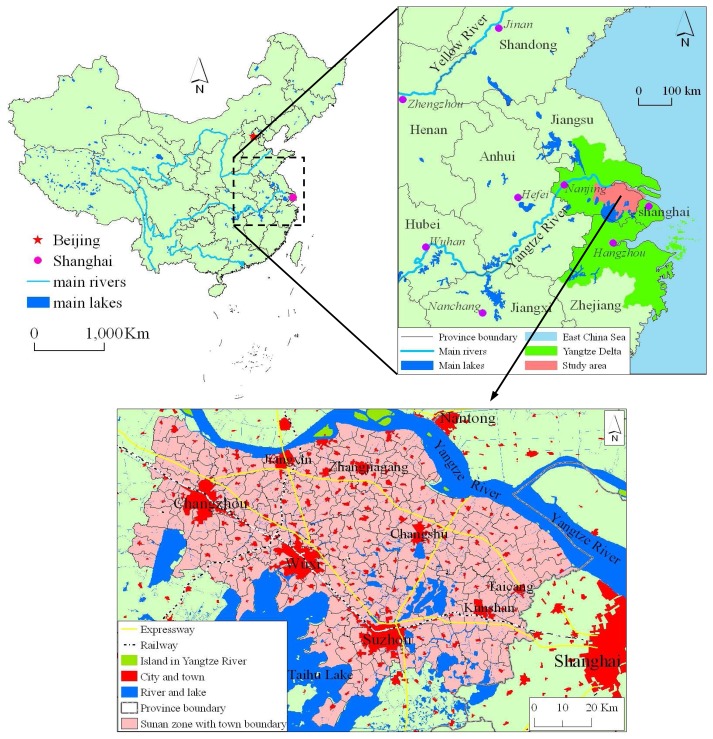
Location of the study area in China.

China's urbanization is going into a fast development stage, and its urbanization level has already reached 43.9% in 2006. Sunan, the southern part of Jiangsu province in the Yangtze River Delta in China, with an area of about 9,200 km^2^, is one of the developed areas with the most rapid urbanization growth in China. It is located on the south bank of the Yangtze River (YR), near the core of the Yangtze River Delta (YRD) (Figure 1).

Sunan's urbanization mode is typical of Chinese urbanization trends. Its rate of urbanization had already reached 67% in 2006. In the initial stage of the “reform and opening-up period” the number of cities in Sunan has risen from three to eight, among which there are three big cities (Changzhou, Wuxi, Suzhou) with a population of more than 5 million, now considered satellite cities of the extended metropolitan region (EMR) of Shanghai [1]. There are five other cities (Jiangyin, Zhangjiagang, Changshu, Kunshan, Taicang) with a population of more than 0.4 million and 205 towns with a population between 10 thousand and 0.4 million.

Within cities, the urban mass distributions in space are never uniform because of differences in land prices and accessibility, for example. Nevertheless, this fragmented distribution within a certain space is not purely random, since fractal objects are structured following a central organization principle, self-similarity throughout the spatial scales, which is a property especially useful for urban geography studies. However, it is possible to neglect the patchwork of intra-urban patterns and their differences when we consider the scale of the whole agglomeration in order to examine the overall sprawl process and patterns in several time periods. A sprawling pattern usually shows a highly irregular form, so it is necessary to find some suitable approaches to describe this pattern, via fractal investigation and spatial autocorrelation in this case.

Fractal properties can be related to some important urban morphology features: self-similarity in clustering and fragmentation of spatial patterns at different scales, hierarchy, sinuosity of borders, and non-linear dynamics. Therefore, some aspects of urban growth are in complete agreement with the fractal description of towns [2] and the fractal is generally quite suitable for describing urban patterns [3]. Basic work on fractal investigation of urban patterns has been done since the 1980s, especially by Batty and Longley [4] as well as by Frankhauser [5]. More recent publications have deepened the methodological applications and confirmed its interest [6-9]. A comparative analysis had been done among some European cities based on fractal measurements [3], which validated the potential of fractal approach.

In the Sunan region, as Figure 1 shows, there is a major urban cluster [10], a group of towns located closely to each other and connected by strong socio-economic linkages. Towns with closer proximity interact more frequently, but vary within their different urban size hierarchy. Urban built-up areas show the extent of urban spaces to a certain extent, and so the built-up areas could characterize the spatial autocorrelation property, which may uncover the urban spatial pattern. Accordingly, spatial autocorrelation measures including Moran I, Getis-Ord G [11], indicating the probability of similar elements being located closely to each other, could be used to quantitatively measure urban sprawl pattern. They have been used widely in epidemiology [12], regional economy [13, 14], floating population [15], criminology [16, 17] and regional society and politics [18, 19]. From the perspective of physics, urban aggregations, such as cities surrounded by satellite towns and commercial areas or larger merged megacities, can be described as special kinds of clusters that can be mapped on a two-dimensional surface.

This paper focuses on clusters of the developed area (built-up area with the following special definition), and aims to uncover urban sprawl pattern of this developed area of China, via quantitative analysis. No attempt has been made to discriminate between various types of buildings in the developed clusters. Section 2 gives the acquisition of object data and the introduction about the study area. Section 3 presents the methodology including fractal dimensions and spatial autocorrelation measure indices, i.e. global and local Moran I, global and local Getis-Ord G. Section 4 presents the results. And some interesting discussions are given in Section 5, linking these measures to changes in Chinese urbanization in recent decades.

## 2. Data

Measures of area coverage and spatial distribution are indispensable to describe the morphology of an urban area adequately [20]. First of all, we need to define urban area and what kind of data to be employed. The definition of “urban area” is controversial since different countries have different means to define “urban” and “urban area” [21]. In principle, “urban area” includes all the land use types that serve the urban function. However, how to classify the urban land use types is often problematic and subjective. It is common that many cities have large areas of water bodies (e.g. lakes and rivers passing through), green lands (e.g. hills and green infrastructure). Additionally, a large number of industry development zones or economic and technical development zones may have been established close to a city or town, which increases the difficulties in defining the urban area. In order to focus on the urban cluster sprawl but not the inner urban structure, we defined the urban area according to the following criteria: (a) if the open space, including water bodies and green lands, is completely surrounded by other urban land use types (e.g. residential, industrial and commercial etc.), it is designated as an urban area and deemed contained within the urban boundary; (b) if an industry development zone or economic and technical development zone is very close to a city or town, its boundary, containing the built-up areas and non built-up areas surrounded by the built-up area, is merged into the urban boundary.

**Figure 2.**
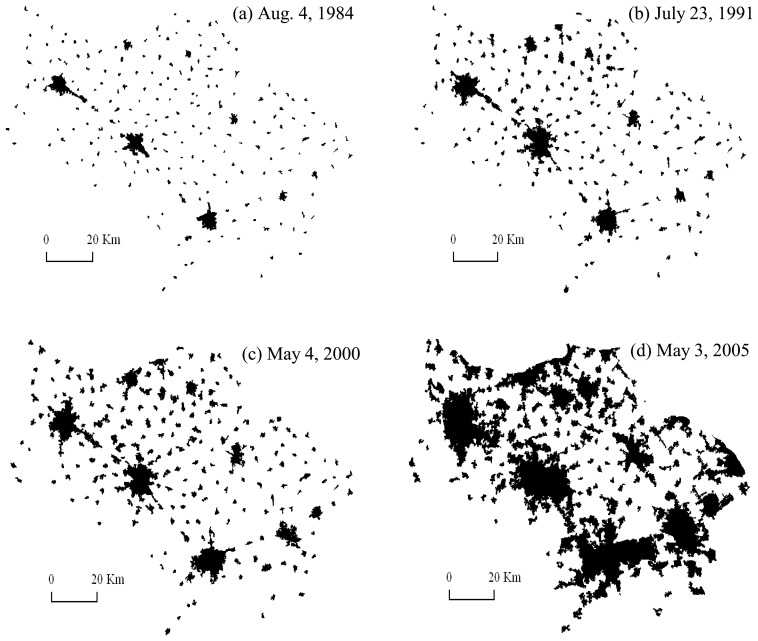
The urban morphology of Sunan in different periods.

Satellite images offer the historical footprint of urban sprawls at certain times, which make the sources of the data derivations comparable [22]. Landsat MSS/TM/ETM images are characterized by medium spatial resolution (about 30 m), affordable and of good quality, easy to access, therefore they are employed mainly in this research. The professional remote sensing images package, ERDAS 8.5, is utilized for image processing. ArcGIS 9.0, one of the most popular and powerful GIS software package, is employed for editing the raw classification data and the subsequent calculation on spatial indicators. The two level product images, acquired respectively in 1984 (August 4), 1991 (July 23), 2000 (May 4), and 2005 (May 3), were selected and then geometrically rectified with 41∼45 control points to the topographic map (scale: 1:50,000, spheroid: Krasovsky 1940, projection: Gauss-Krüger) by using the nearest neighbor method. RMS error (RMSE) was maintained less than 0.5 pixel. Some image enhancement approaches, e.g. LUT stretch and texture enhancement, were used to sharpen the different object boundaries. Then visual interpretation was employed to extract urban boundary *via* the following procedures:

(a) taking the 1:50,000 scale topographic map from the 1980s as reference, the urban boundaries were digitized based on the 1984 Landsat MSS image (Figure 2a);
(b) taking the boundaries in 1984 as reference, the boundaries in 1991 were digitized based on the Landsat TM image in 1991 (Figure 2b);
(c) taking the boundaries in 1991 as reference, the boundaries in 2000 were drawn out based on the Landsat ETM image in 2000 (Figure 2c);
(d) taking the boundaries in 2000 as reference, the boundaries in 2005 were outlined on the basis of the 23 m spatial resolution IRS-P6 multi-spectral image acquired on May 3, 2005 (Figure 2d).
(e) Finally, for the analysis of fractal dimension, the urban area for each town was exported into a black and white color 4,724 × 4,724 pixel bmp file (each pixel 250 m × 250 m), respectively, representing the urban area and non-urban areas.

## 3. Methodology

### 3.1 Fractal dimension

Fractal geometry has become popular through the work of the mathematician Mandelbrot [23]. Fractal dimension measures are a good instrument for a global comparison of the morphology of cities [2]. In this section, we give more details about fractals through description of how to determine the fractal dimension, including radius dimension, correlation dimension, and boundary dimension. The first three dimensions attribute to the counting method, which was implemented by Fractalyse developed by Gilles Vuidel. The fourth can be calculated by the regression function in a statistics software package.

The counting method goes step by step in an iterative principle. In each iterative step, the method involves counting the number of black pixels contained in a counting window. From one step to the next, the size of the counting window is enlarged. In doing so, we artificially change the level of analysis of the image. Thus, for each method we have two elements varying according to the counting step (iterative step): (a) the number of counted elements (which is roughly the number of black pixels present in the window) (*N*); and (b) the size of either the counting window or the reference element (*ε*). Then, we obtain a series of points that can be represented on a Cartesian graph. The Y-axis corresponds to the number of counted elements (*N*) and the X-axis corresponds to the size of the counting window or that of the reference element *ε*, with *ε* increasing from step to step. Mathematically, the series of points is a curve (named the empirical curve). The next stage is to fit this empirical curve with another one, the estimated curve. If the empirical curve follows a fractal law, the estimated curve has the form of a power law (parabolic or hyperbolic), and *D* represents the fractal dimension [24].

(1) $$N={ɛ}^{D}\text{or}\;N={ɛ}^{-D}$$

Generally speaking, the image is not a pure fractal, i.e. not a continuous function but a discrete and finite one, so it is only possible to approximate the fractal law. Therefore, we do not estimate directly the fractal law as equation (1) but a generalization of it as follows,

(2) $$N=a{ɛ}^{D}+c$$

where, *a* is called the “pre-factor of shape”. It gives a synthetic indication of the local deviations from the estimated fractal law. In the case of a mathematical fractal structure *a* should be equal to 1. In some cases *a* is equal to 0.5 or 3. If its value goes over 10 or beyond 0.1 the fractality of the structure under study is not confirmed [2]. *c* corresponds to the point of origin on the Y-axis.

The distinction between different types of urban patterns was first discussed in Frankhauser [5] and Batty and Longley [4]. Batty and Xie [25] and Shen [26] did some research on comparative analysis. In this paper, a non-linear regression is used to find approximately the power law which best fits the empirical curve. The quality of the estimation is quantified using a correlation coefficient: if the correlation coefficient (an output of Fractalyse) is less than 0.999, the computed fractal dimension is considered to have a poor adjustment to the theoretical curve, if it is inferior to 0.9999 we have a good adjustment, if it is in the range between 0.9999 and 1.000000 we have an excellent adjustment. If the fit between the two curves (empirical and estimated ones) is bad, two conclusions are possible: either the pattern under the study is not of a fractal nature or it is of a multi-fractal nature. In order to measure the morphological evolution of the urban area of Sunan we used all of the three methodological approaches mentioned above to provide complementary insights on the fractality of the urban patterns from different views, which have different geographical meanings. Additionally, the curve of scaling behavior *α*(*ε*), highlighting the presence of a constant value of the parameter α and of increasing or decreasing trends of the represented curve and allowing the characterization of the ranges of the radius in which the fractal dimension is constant with a good correlation coefficient, will help us identify the threshold at which the urban cluster morphology may change from one point to another. Three methods are employed:

#### a. Radius method

This method refers to a specific point known as the counting centre and gives the law of distribution of the occupied sites around this point. The radius dimension (*D_r_*) indicates that the attenuation features of spatial distribution departing from the center of the analysis window to its periphery. *D_r_* may not exceed the value of 2 in theory. If *D_r_* is less than 2, it shows that spatial distribution of the town attenuates in density from the center to its periphery; if *D_r_* is equal to 2, it shows the spatial distribution of town system element is uniform to the direction of radius. In real world patterns the local values of the scaling exponent, as represented by the curve of scaling behaviour, may indeed exceed two, which could be explained in detail in Frankhauser [27]. In this study, we choose the counting centers, respectively, at the Sunan's baricenter (310, 335), Changzhou (113, 229), Wuxi (253, 322), and Suzhou (388, 441), where mass is concentrated and its density is not basically augmented when increasing distance from the counting centre.

#### b. Correlation method

Each point of the image is surrounded with a small squared window. The number of occupied points inside each window is enumerated. This allows the mean number of points per window of that given size to be calculated. The same operation is applied for windows of increasing sizes. In principle it is possible to choose any shape for the window, such as circle, hexagon, etc. However, since pixels are square-like, the choice of a square helps to avoid rounding errors. The correlation dimension (*D_c_*) shows uniformity degree of urban distribution in a certain area; and it gives a detailed result about the distribution of occupied points. Generally, *D_c_* is in the range of 0 to 2. If *D_c_* is more close to 2, it shows all of towns distribute more uniformly over space; and if *D_c_* is more close to 0, it shows one premier city has been formed.

#### c. Boundary method (or area-perimeter method)

If those urban surfaces are simple geometrical objects, their borders would be characterized by the dimension 1 and their surfaces by the dimension 2. Although the observable relation between borders and surfaces is regular, the ratio surface to border is about 1.05 [2, 5], which contradicted with Euclidean geometry but corresponds to fractal geometry. Generally for each of urban polygons, its perimeter *P* is related to the area *A* of the same polygon by the basic fractal relationship [28]:

(3) $$P=kA^{D/2}$$

where *D* is the fractal area-perimeter dimension (*D_a_*), k is the constant of proportionality. Equation (3) can be transformed logarithmically:

(4) $$\ln A=\frac{2}{D_{a}}\times \ln P+c$$

where *c* is the intercept (constant) for linear regression. We employed ARCGIS 9.0 for *A* and *P*; and the statistic software SPSS 11.0 was employed for regression to acquire the value of *D_a_*. Similarly, the quality of the estimation is quantified using a correlation coefficient. In general, *D_a_* is in the range of 1 to 2. But recent investigations showed that the situation might be more complex. Indeed for urban patterns often intermediate situations are observed, and no significant relation has been detected between the fractal dimension of surface (*D_surf_*) and the dimension of boundary (*D_bound_*) when comparing town sections [24]. However, from a theoretical point of view, for Sierpinski carpets and Fournier dusts there exists indeed the relation *D_surf_* =*D_bound_*, but for teragons it may shown *D_surf_* =2 and 1< *D_bound_* <2 [29].

### 3.2 Compactness index

We employ the indicator compactness derived from “landscape metrics” to quantitatively measure the urban form. Compactness not only measures the patch shape for the individual patch, but also considers the dispersion degree of the landscape. The compactness index (*CI*) defined by Li and Yeh [30] is:

(5) $$\text{CI}=\frac{\sum_{i}P_{i}/p_{i}}{N}=\frac{\sum_{i}2\sqrt{S_{i}/\pi }/p_{i}}{N}$$

where *S_i_* and *p_i_* are the area and perimeter of patch (here, urban area), *i*, *P_i_* is the perimeter of a circle with the area of *S_i_* and *N* the total number of patches. According to this definition, the compact patch with the round shape will have the high value. To minimize the bias caused by the numerous small compact patches rather than the large complex ones. Li and Yeh [30] also revised compactness index as follows:

(6) $$CI\prime =\frac{CI}{N}=\frac{\sum_{i}2\sqrt{S_{i}/\pi }/p_{i}}{N^{2}}$$

### 3.3 Sprawl intensity

In addition to static analysis of urban forms by a fractal dimension at some time and dynamic analysis by the evolution of the fractal dimensions in the course of time, it is necessary to select a dynamic index for showing the urban and urban cluster growths more directly. So we employ the sprawl intensity index (*SII*) as the following:

(7) $$\text{SII}=\frac{A_{s}}{A_{t}\times \Delta t}\times 100$$

where *A_t_* (in m^2^) is the total area within the administrative town boundary, and *A_s_* (in m^2^/year) the urban sprawl area of each of the towns along some direction or directions in its corresponding administrative boundary during the period time Δ*t*(in years). In China, the town is the most basic administrative unit, whose boundary is usually merged or split partially or wholly according to its economic development at that time. The boundary may not be the same in different periods. Here, we take the administrative boundaries in 1991 for the basic calculation and analysis unit for their convenient comparisons in the course of time.

### 3.4 Spatial autocorrelation

Some standard global and new local spatial statistics, including the Moran I [31], Getis-Ord G [32], and Local Indicators of Spatial Association (LISA) [33], can be employed to detect the sprawl pattern of urban cluster [34]. They start from the assumption of a randomized distribution of spatial pattern. Or the spatial pattern or form for the spatial dependence is derived from the data only without preconceived theoretical notion. In this study, the global and local Moran I were carried out by GeoDa 0.9.5-i (Beta) developed by Luc Anselin; and the global and local G statistics were calculated by Spatial Statistics Tools in ArcGIS 9.0.

#### a. Global Moran *I*

The Moran *I* is defined by

(8) $$I=\frac{n}{S_{0}}\frac{\sum_{i}^{n}\sum_{j\neq i}^{n}w_{ij}(x_{i}-\bar{x})(x_{j}-\bar{x})}{\sum_{i}^{n}{\left(x_{i}-\bar{x}\right)}^{2}}$$

where *n* is the number of observations, *x_i_* and *x_j_* denote the observed value (of sprawl intensity in this study) at location *i* and *j*, respectively, *x̅* is the average of the {*x_i_*} over the *n* locations, *w_ij_* is a symmetric binary spatial weight matrix (*n*×*n*) defined as 1 if location *i* is contiguous to location *j* or location *i* and *j* are within a certain distance *d* and 0 otherwise, and *S*_0_ is the sum of all the elements from *w_ij_*.

The value of Moran *I* ranges from -1 to 1. The Moran *I* is positive when the observed value of locations within a certain distance or their contiguous locations tend to be similar, negative when they tend to be dissimilar, and approximately zero when the observed values are arranged randomly and independently over space.

#### b. Global Getis-Ord *G*

The Getis-Ord *G* is defined by

(9) $$G(d)=\frac{\sum \sum w_{ij}(d)x_{i}x_{j}}{\sum \sum x_{i}x_{j}}$$

where the symbols have the same meaning as in equation (8). For ease of interpretation, a standardized form of *G*(*d*) can be defined as:

(10) $$Z(G)=\frac{G-E(G)}{\sqrt{\text{Var}(G)}}$$

where *E*(*G*) is mathematical expectation of *G* and *Var*(*G*) variance of *G* If *G* is more than *E*(*G*) and *Z*(*G*) is significant, the observations are clustered by relatively large values; if *G* is less than *E*(*G*) and *Z*(*G*) is significant, the observations are clustered by relatively small values; and if *G* is close to *E*(*G*), the observations are randomly distributed over space.

Each of the two statistics mentioned above only gives a single value to show a whole spatial pattern for observations, so we cannot know about the spatial variance at each of locations. Additionally, the global statistics is on the hypothesis of the mathematical expectation and variance of all the observations being a constant; however in fact, it is impossible for satisfying the hypothesis especially when the data volume is enormous. In a global spatial autocorrelation samples, there may be randomly distributed observations in local locations; or in a global randomly distributed observations, there may be a local spatial correlation pattern. So it is very necessary to employ local statistics to identify the local spatial pattern.

#### c. Local Moran *I*

The local Moran statistic for each observation *i* is defined as:

(11) $$I_{i}=\sum w_{ij}\;Z_{i}\;Z_{j}$$

where the observations *Z_i_* and *Z_j_* are in standardized form (with mean of zero and variance of one). The spatial weight *w_ij_* are in row-standardized form. So, *I_i_* is a product of *Z_i_* and the average of the observations in the surrounding locations. The value of *I_i_*, unlike that of global Moran *I*, is tightly related with the observations, and not confined to the range of -1 to 1.

With a significant level (such as *p*-value less than 0.05), a positive *I_i_* and a positive *Z_i_* indicate that a high observation value at location *i* is associated with relatively high values at its surrounding locations, viz. high-high value cluster (HH); a positive *I_i_* and a minus *Z_i_* indicates that a low observation value at location *i* is associated with relatively low values at its surrounding locations, viz. low-low value cluster (LL); a minus *I_i_* and a positive *Z_i_* indicates that the observation value at location *i* is much more than those at its surrounding locations, viz. high-low value cluster (HL); and a minus *I_i_* and a minus *Z_i_* indicate that the observation value at location *i* is much less than those at its surrounding locations, viz. low-high value cluster (LH).

#### d. Local Getis-Ord *G*

The global Getis-Ord *G* may not easily distinguish the presence of negative spatial association from spatial clustering, which is often defined as either high-rate or low-rate spatial clustering. The global *G* has not been evaluated extensively, especially for low-value clustering. It is critical to interpret local *G* according to the degree of the global *G*[35]. The local *G* (including *G_i_* and $G_{i}^{*}$) is to test the deviation of a local pattern from the average values of observations. The spatial statistic *G_i_*(*d*) and $G_{i}^{*}(d)$ can be defined as:

(12) $$\begin{array}{cc} G_{i}(d)=\frac{\sum_{j,j\neq i}^{n}w_{ij}\;(d)x_{j}}{\sum_{j,j\neq i}^{n}x_{j}} & \;G_{i}^{*}(d)=\frac{\sum_{j}^{n}w_{ij}\;(d)x_{j}}{\sum_{j}^{n}x_{j}} \end{array}$$

where the symbols are the same as before. For easy interpretation, a standardized form of *G_i_*(*d*) in Ord and Getis [36] can be defined as:

(13) $$\begin{array}{cc} Z(G_{i})=\frac{G_{i}-E(G_{i})}{\sqrt{\text{Var}(G_{i})}} & \;Z(G_{i}*)=\frac{G_{i}*-E(G_{i}*)}{\sqrt{\text{Var}(G_{i}*)}} \end{array}$$

where *E*(*G_i_*) is mathematical expectation of *G_i_* and *Var*(*G_i_*) is the variance; and $E(G_{i}^{*})$ is mathematical expectation of $G_{i}^{*}$ and $\text{Var}(G_{i}^{*})$ is the variance.

A significant and positive *Z*(*G_i_*) or $Z(G_{i}^{*})$ indicates that the location *i* is surrounded by relatively large values, whereas a significant and negative *Z*(*G_i_*) or $Z(G_{i}^{*})$ indicates that the location *i* is surrounded by relatively small values, so the local *G* statistics can be used to identify spatial agglomerative patterns with high-value clusters or low-value clusters. Having shown the methodology of this study, we now turn to the results of the analysis.

## 4. Results

### 4.1 General situation

**Figure 3.**
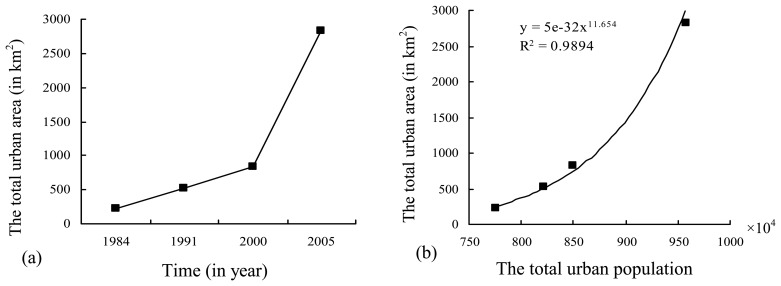
The total urban areas in different years and the relationship with the corresponding total urban population.

In the period from 1984 to 2000, the urban area became linearly larger and larger from about 230 km in 1984 to 750 km^2^ in 2000; then it suddenly sped up exponentially to about 2,800 km^2^ in 2005 (Figure 3a), with 900 km^2^ covered by various development zones including industry development zones and economic and technical development zones. The total urban area of the Sunan zone in 1991 is 2.33 times that of 1984, in 2000 it is 1.57 times that of 1991 and 3.64 times that of 1984, in 2005 it is 3.41 times that of 2000, 5.34 times that of 1991 and 12.42 times that of 1984. The relationship between the total urban area and the total urban population agrees greatly with a positive exponential function (Figure 3b). And the urban area growth is much faster than the urban population growth, which means that the urban growth is land-enclosed.

### 4.2 Homogeneity and compactness

**Figure 4.**
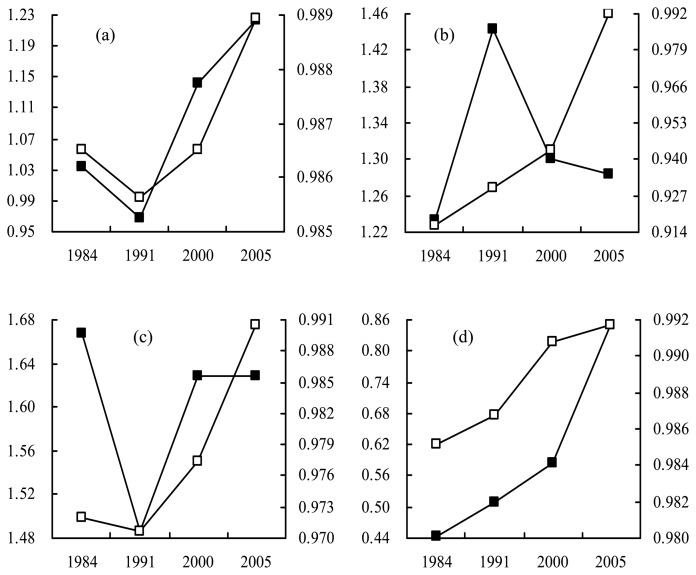
Global fractal radius dimensions and their corresponding correlation coefficients centered at: (a) barycenter, (b) Suzhou, (c) Wuxi, and (d) Changzhou; left y-axis and solid black rectangle for dimension (dimensionless), right y-axis and hollow white rectangle for correlation coefficient (dimensionless), *x*-axis for time (in year).

**Figure 5.**
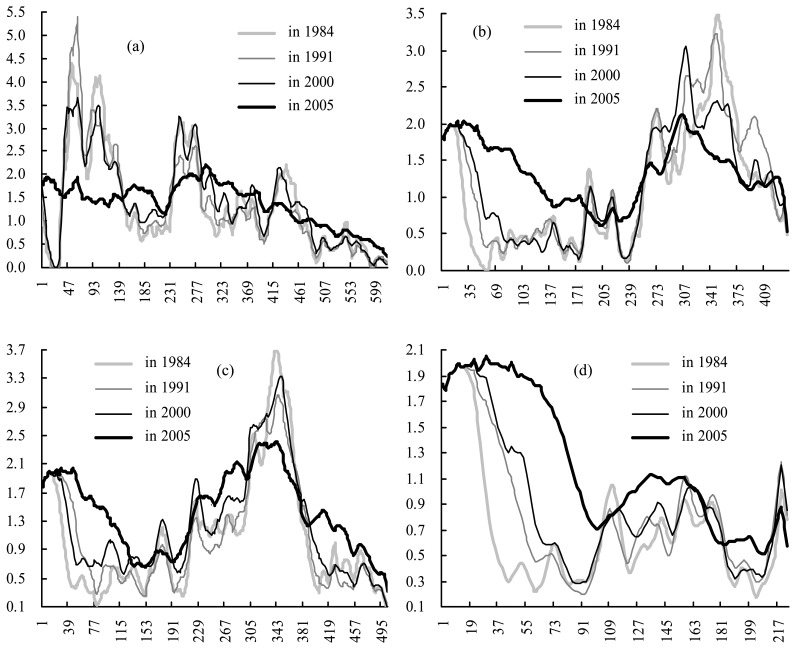
Scaling behavior in the five years, respectively, centered at: (a) baricenter, (b) Suzhou, (c) Wuxi, and (d) Changzhou; y-axis for *α* (dimensionless), *x*-axis for *ε* (in pixel).

The global fractal radius dimension (GFRD) centered at the baricenter (310, 335), with a maximum effective circle radius range of 1 to 619 pixels containing almost all the towns, shows on the whole that the repartition of urban areas has become more homogeneous over time except in 1991 with a value below 1 (Figure 4a), revealing that the spatial organization is like a Fournier's dust in 1991. A similar tendency with big and many oscillations (meaning different reliable local dimensions with many estimated intervals) is illustrated by scaling behavior curves in 1984, 1991 and 2000 (Figure 5a), reveals similar heterogeneous spatial organization of urban surfaces, and a strong dilution existed in the radius range of 150 to 230 pixels. With urban sprawl, the curve in 2005 was obviously different from the others; it reveals more a homogenous distribution and is more compact, especially in the radius range of 1 to 400 pixels.

GFRD centered at Suzhou (388, 441), with a maximum effective radius circle range of 1 to 441 pixels containing Wuxi, Kunshan, Taicang and Changshu, implies to a certain extent homogenization of spatial organization of the urban area from the center to its periphery over time from 1984 to 2005 (Figure 4b); however, the value in 1991 was evidently more than the others, which shows more homogeneity. But from the view of different estimated intervals (Figure 5b), the scaling behavior curves in 1984, 1991 and 2000 illustrate a similar trend towards urban sprawl, indicating the similar spatial organization around Suzhou along the radius from center to periphery and a similar dilution radius range of about 50 to 170 pixels, far away from Wuxi and Kunshan, with a high correlation coefficient more than 0.99; however, in 2005, the dilution radius range were extended to about 140-240 pixels, just reaching the outline of Wuxi and Kunshan, with a local fractal radius dimension (LFRD) of 0.0011 and a correlation coefficient of 0.999120.

GFRD centered at Wuxi (253, 322), with a maximum effective circle radius range of 1 to 505 pixels containing Suzhou, Changzhou, Jiangyin, Zhangjiagang and Changshu, shows more homogeneity than that centered at Suzhou in the four years, especially in 1984, 2000 and 2005 (Figure 4c), implying a different spatial organization of urban areas from the centers of Suzhou and Wuxi from their respective peripheries. It is worth noting that the GFRD in 2000 was almost equal to that in 2005 and obviously more than that in 1991, revealing that the urban sprawl process is becoming more and more metrical, and at present is relatively stable. Scaling behavior curves uncover there is a similar tendency with increasing ε (Figure 5c): in 1984, 1991 and 2000 existed a dilution radius range of about 50 to 160 pixels, far way from Suzhou and Changzhou, with a LFRD of less than 1 and a high correlation coefficient more than 0.99, however in 2005, which became a dense and narrowing radius range of 125 to 200 pixels, just reaching the outline of Suzhou and Changzhou, with a LFRD of 1.489 and a correlation coefficient of 0.999411.

When the center is transformed into Changzhou (113, 229) with a maximum effective radius circle range of 1 to 225 pixels, there are higher and higher values of GFRD but all less than 1 over time (Figure 4d), showing evident heterogeneity and Fournier's dust characteristics in each of the four years. Maybe this is due to its fringe location in the whole study area. Scaling behavior curves show that the outline is extending towards its surrounding, and so does the dilution radius range up to nearly reach the outlines of Jiangyin and Wuxi.

**Figure 6.**
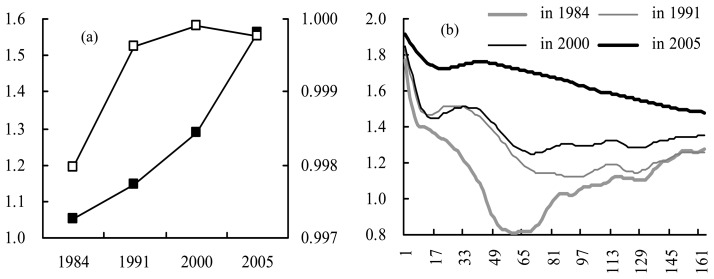
(a) Fractal correlation dimension, left y-axis and solid black rectangle for dimension (dimensionless), right y-axis and hollow white rectangle for correlation coefficient (dimensionless), x-axes for time (in year); (b) the scaling behavior, y-axis for *α* (dimensionless), *x*-axis for *ε* (in pixel).

In 1984, the global fractal correlation dimension (GFCD) was close to 1 (Figure 6a) and there were also big fluctuations of dimensions from about 0.55 to 1.43 for different estimation intervals (i.e. the local fractal correlation dimension, LFCD) (Figure 6b), showing heterogeneous spatial organization like Fournier' dust especially in the ε ranges of 23 to 58, 58 to 69, 69 to 86, 118 to 130 and 130 to 154 pixels because of LFCD less than 1 with correlation coefficients more than 0.999. This corresponds to the towns which are distant from each other. GFCD were higher (less than 1.3) in 1991 and 2000 but keep similar values for different estimation intervals and the largest difference was in 1991 that there were still some LFCD less than 1 with ε ranges of 28 to 90 and 112 to 126 pixels. The fractal correlation dimension in 2005 was highest (more than 1.5) in the different estimation intervals, which means the urban area has become more homogeneous.

**Figure 7.**
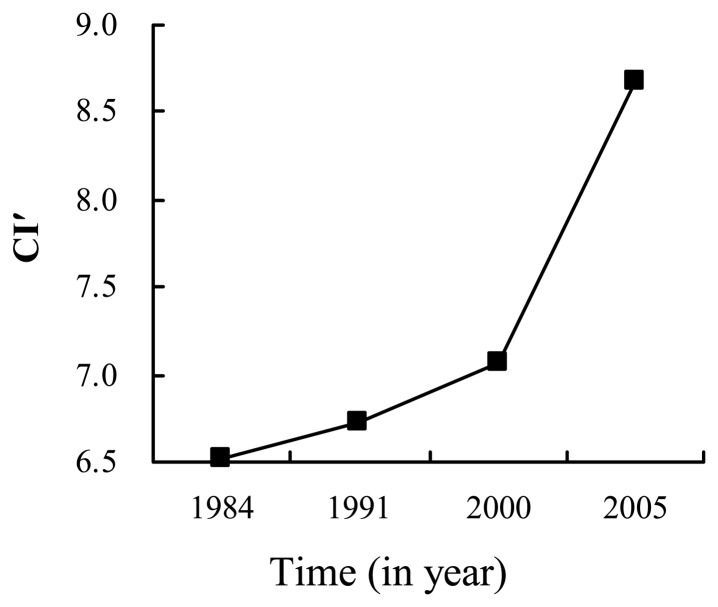
The revised compactness indices.

**Figure 8.**
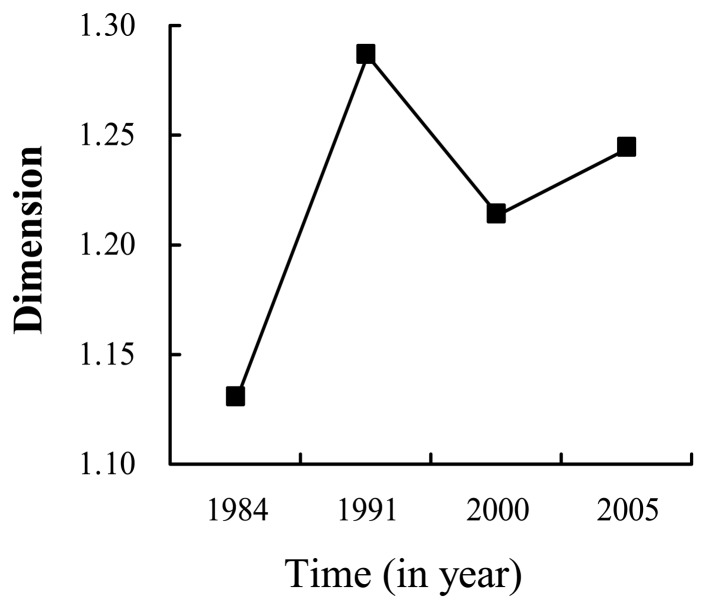
The fractal boundary dimensions.

Figure 7 illustrates that *CI*′ became higher and higher over time from 1984 to 2005. It reveals on the whole that the connection between towns is more and more compacted, which validates the analysis from fractal dimension. Different from the implication of the fractal dimensions and the revised compactness indices, i.e. the urban surface becoming more and more homogenous and compact, the comparison of fractal boundary dimensions shows on the whole that the outlines of urbanized surfaces are unstable and irregular (Figure 8). It reveals wholly that urban outline is to some extent out-of-order from 1984 to 2005, it may due to the lack of a continuous urban planning schema over a long time even though there has been some urban planning during some time periods.

### 4.3 Sprawl pattern

Means of *SII* of all the towns are 0.54 during the periods from 1984 to 1991, 0.34 from 1991 to 2000 and 3.65 from 2000 to 2005 respectively, showing that the sprawl intensity increased sharply in the new century and is about 6.8 times that in the initial stage of reform and opening-up period, after going through a transitional stage from 1991 to 2000.

The sprawl is clustered to a certain extent on the whole in each of the three periods, revealed by the global Moran I of *SII*, the spatial weights of which were constructed based on contiguities, respectively, from polygon boundary files (*I* = 0.427 in 1984-1991, 0.176 in 1991- 2000, 0.294 in 2000-2005), from the average nearest neighbors with a threshold distance of 5,000 m (the nearest neighbor observed mean distance is about 4,910 m, calculated in ARCGIS 9.0) and from the nearest neighbors with a threshold distance of 10,000 m (Table 1). On the whole, the clustered degree is the highest during the period of 1984-1991, followed by 2000-2005, and the lowest in 1991-2000, which show that: (a) in 1984-1991, urban sprawl intensity was very heterogeneous and the prominent sprawl might happen only around several towns or cities; (b) in 1991-2000, urban sprawl intensity became a little homogeneous; and (c) in 2000-2005, the towns grew heterogeneously again, but the prominent sprawl happened around more towns or cities.

**Figure 9.**
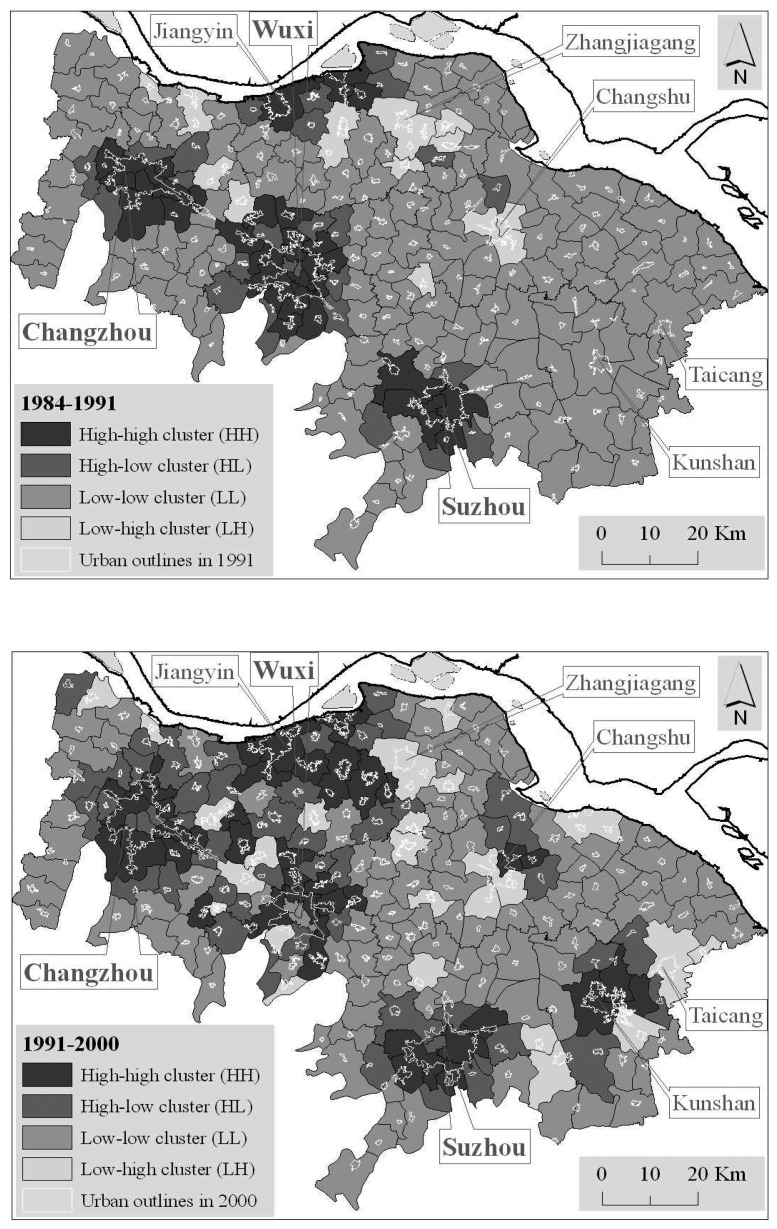

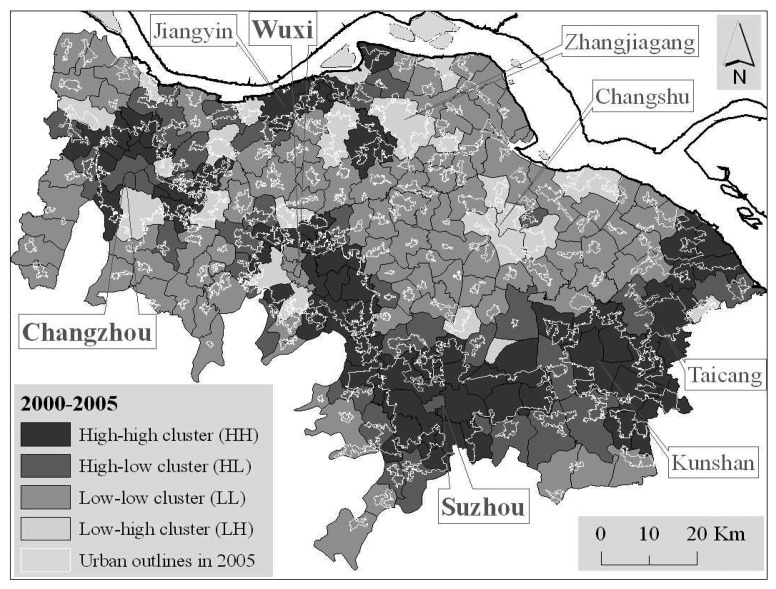
Moran I scatter map.

However, the clustered patterns indicating urban sprawl were different, which were confirmed by the local Moran *I* of *SII*. The local Moran *I* scatter maps of *SII* show that (Figure 9): (a) There are obvious HH, HL, LH and LL clusters with similar spatial distribution, but different sizes in the three periods; some towns around Changzhou, Wuxi and Suzhou were classified into the HH cluster in each of the three periods and some towns along the Yangtze River were always in the HH cluster region; additionally, HH cluster regions were gradually transformed from the city cores to the suburbs during the periods from 1984-1991 to 2000-2005. (b) In 1984-1991, HH clusters were focused on towns associated with the three cities of Changzhou, Wuxi and Suzhou and several towns

Along the Yangtze River, around which several HL clusters existed, the majority of towns were classified into the LL cluster. It reveals that urban fast growth was mainly centralized in the three big cities during this period. (c) In 1991-2000, HH clusters were extended in spatial distribution; there were more and more HH cluster towns along the Yangtze River, and towns subjected to Kunshan were classified as HH cluster but as LL cluster in 1984-1991. (d) In 2000-2005, the HH clusters were contiguously joined into a zonal region from Wuxi, through Suzhou and Kunshan, to Taicang; additionally, the suburbs were in HH cluster like in 1991-2000. (e) Wholly and generally, HH cluster was point-pattern at the initial stage, and then they were transformed into the periphery or enlarged into a large region but still point-pattern; with rapidly developing economy, more HH clusters emerged and some of them were joined together into a zonal region.

In order to uncover different clustered patterns more deeply and formally, the hot/cold spot analysis technique was employed to calculate the global Getis-Ord *G* values. The global Getis-Ord *G* value, together with the *E* value and *Z* score (Table 2), show that the cluster revealed by the global Moran *I* is a high cluster, which is more significant in 1984-1991 than in the other two periods. So the hot spot of urban sprawl was highly concentrated in 1984-1991, and was gradually dispersed. In order to uncover the spatial distribution of the hot spots and their transformation, the local Getis-Ord *G* values were calculated as well.

## 5. Discussion

Urban form is a “pattern”, representing the spatial characteristics of the urban area at a certain time. Urban form is also a “process”, indicating the spatial change over time. The pattern is the outcome of the process. And both the pattern and process are closely linked to social, economic, cultural and other factors [37].

**Figure 10.**
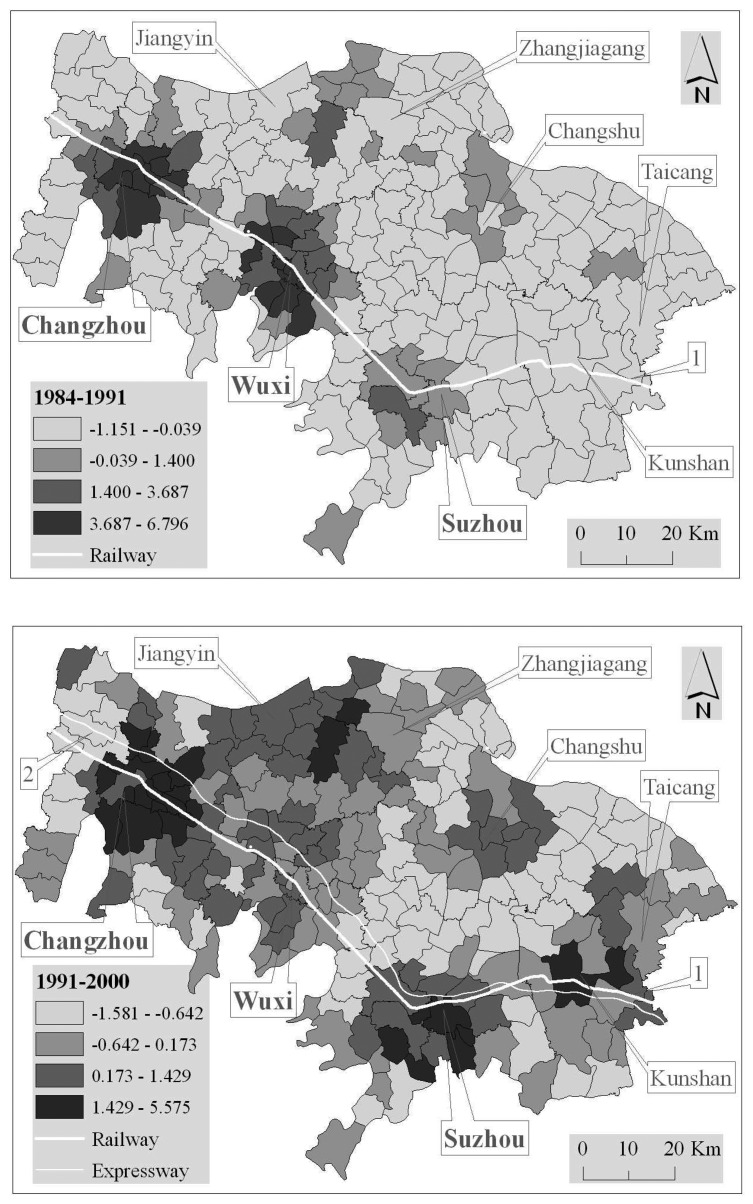

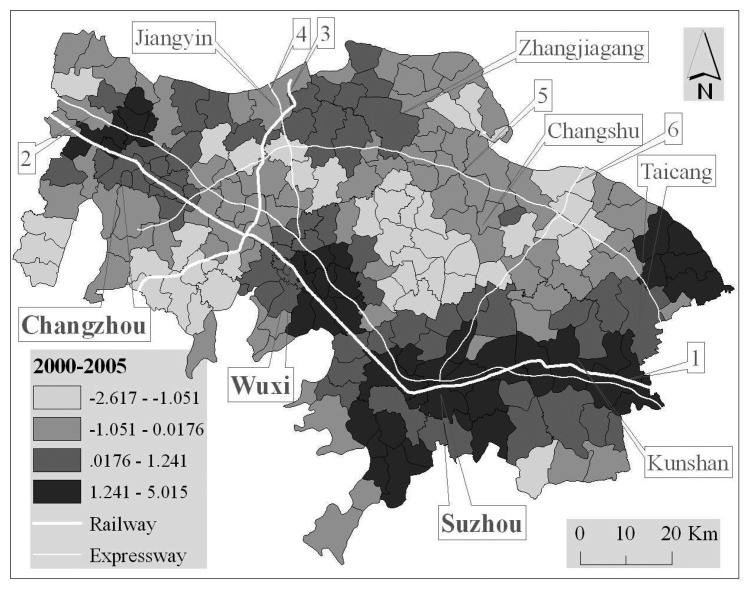
Spatial distribution of the local Getis-Ord *G*; 1 denotes Huning railway, 2 Huning expressway, 3 Subei railway, 4 Xicheng expressway, 5 Yanjiang expressway, and 6 Sujiahang expressway.

Figure 10 shows from the sprawl intensity point of view that:

(a) In 1984-1991, there were four hot spots, which were concentrated, respectively, at the four cities, i.e. Changzhou, Wuxi, Suzhou and Jiangyin. The former three are connected directly by the Huning railway (from Nanjing to Shanghai) and the fourth lies along the Yangtze River;
(b) In 1991-2000, the hot spots located at Changzhou and Jiangyin, respectively, still existed but dispersed into a big connected patch; the hot spot located at Suzhou was enlarged and a new one grew up at Kunshan; the one located at Wuxi became weaker;
(c) In 2000-2005, the hot spot located at Changzhou still existed, but was becoming weaker and weaker, so did the big connected patch; the one located at Wuxi was enhanced again; noticeably, a zonal hot spot had grown up from Wuxi, through Suzhou, to Kunshan along Huning railway and Huning expressway; additionally, a new one emerged at Taicang along the Yangtze River;
(d) Wholly and generally, the hot spots of urban sprawl were concentric mainly at big cities in the initial stage, where the urban sprawl were self-governed and did not have strong influence on each other; and then, the hot spots gradually spread to their surrounding towns, or they were joined into other hot spots into a big connected patch; with economic and social development, the hot spots spread and dispersed continuously and some were joined into a zonal region along the important transportation axes.

During the last 25 years, China has gone through three significant development stages, each of which pushed economic and social development, and then urbanization process was sped up to a great extent. The first stage started in the early 1980s, motivated by the “reform and open door” policy of Deng Xiaoping. The agricultural and state-owned economies dominated the national economy in the early days of this period; and then the industrial economy and non-state economy, including the collective economy and individual economy, were developed rapidly due to the beneficial and favourable government policies. The people in the study area enjoyed a better standard of living than those in most of the other areas of China.

In 1984, 14 cities, including Shanghai, were opened up as “opening city in coastal areas”, and some regions, e.g. YRD and the Pearl River Delta, were developed as “special economic zones”. The Sunan zone thus greatly benefited from the policy and the three traditional and historical cities – Changzhou, Wuxi and Suzhou – were impelled to develop prior to other cities and/or towns by this policy. However, the development at this stage were greatly affected and restricted by China's economic development direction of traditional long-standing “planning economy”, and the criteria to judge the development was not made clear yet. The second stage started in 1992, was initiated by Deng Xiaoping's new lecture on the “reform and opening up” policy when he visited South China again. China then started to establish a socialist market economy system with Chinese characteristics under the slogan of “deepening reform and opening up”. The criteria determining the success of the development were whether it would be beneficial to developing socialist productive forces, whether it helps enhancing the overall strength of the country and whether it would increase people's living standards. Under this beneficial policy, more and more towns began to speed up their development by attracting foreign investment with the help of predominant geographical locations, preferential investment policies including land use, duty and revenue, and a good pleasant investment environment including infrastructure preparation, government service and rich human resources. The industrial economy became more and more important and prominent. The latest stage started in 2000 when the population entered the great 21st century, initiated by the significant development targets set for the new century by the central government of China. The foreign investment was continuously surging upwards but began to be controlled by the macroeconomic decision-making process. In this period, the industrial economy has occupied the dominant position in the whole national economy and was increasingly affected by the most economically developed city of Shanghai in China. Shanghai was creating more attraction to its surroundings. This is the reason why we divided the whole study period of 1984 to 2005 into the three stages of 1984 to 1991, 1991-2000 and 2000-2005.

**Figure 11.**
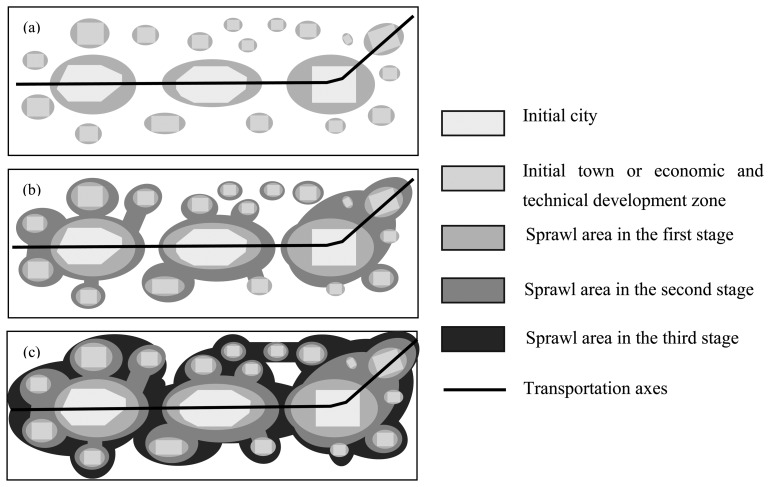
Urban sprawl patterns in the developed area of China at different stages since reform and opening-up: from an individual city to a big urban group or an urban cluster.

Wong *et al.* [38] and Shen [39] argued that Chinese urbanization pattern since reform and opening-up is distinguished by dual-track urbanization, i.e. state-sponsored urbanization and spontaneous urbanization; the former increases non-agriculture population and the latter contributes to small town-based urbanization and migration of floating population. Both tracks contribute significantly to the transformation of the spatial pattern of urbanization [38]. In spatial terms, urbanization behaves as urban growth or sprawl, which is related closely to the developmental level and mode. In turn, the development determines the pattern of urban sprawl. Since 1992, in the study area, a large number of industrial development zones and/or economic and technical development zones, most of which adjoined urban areas closely, were rapidly appearing and growing. This is the main direct cause for why the urban area increased rapidly in the developed area of China, including Sunan zone, within a short period. Although some developments kept away from the urban areas at their initial establishment stages and some gaps existed, the gaps were infilled along the connecting routes to the urban area quickly in a short time. Additionally, with the rapid development of the economy and increase in income, wealthy people can afford private motor vehicles and the public traffic moved farther away from the inner town, which makes it possible for people to live in suburban areas as in most developed countries, e.g. U.S. or Australia. This is also one of the factors of urban sprawl. Thirdly, more people are migrating from villages to cities for more employment opportunities; some of them possess the right of permanent residence as a result of the reform of the strict resident registration system. They choose to live in suburbs farther away from the midtown because of lower income. The suburb is becoming an interlaced place, for wealthy people, employees and peasants to live in, which speeds up the urban sprawl. Fourthly, when the urban center is sprawling gradually from the center to periphery, it fuses the surrounding towns and/or villages (i.e. rural-urban transformation), which, in turn, increases the area of the urban sprawl in a short time. Thus the sprawl reduces the spatial contrast between rural and urban areas. It is probable that the urban form is also affected to a certain extent by information-based variables, e.g. telephone, broadband, internet, etc. [40, 41], but there are different viewpoints on the modes of influence of these factors. Regardless of what reasons, it is a fact in our research that all of the towns sprawl outward to their boundaries, making it possible for them to become more compact and homogenous. Different towns have different geographical characteristics, human environments and different material investment policies, which results in differential urban growth. The important transportation axes, i.e. Huning railway and Huning expressway from Nanjing to Shanghai, play an important role in the course of urban sprawl, which reveals the urban sprawl is selective to the direction. So the urban sprawl pattern can be summarized as follows:

(a) Some big cities benefiting from the preferential policy in the reform and opening-up environment firstly began to grow up at the initial stage, but their sprawls were unconnected to each other (Figure 11);
(b) With the development of economy and the establishment of suitable policies, more and more urban sprawls fused with their surrounding towns, industrial development zones or economic and technical development zones and others; there are more attractions to each other between different cities by more strengthening functions of the important transportation axes or more explicit economic complementarities including city functions (Figure 11b);
(c) The transportation axes are increasingly important in economic development and regional cities are establishing more explicit functional divisions. Gradually, some cities and/or towns were joined together and began to be fused into a big city group or an urban cluster (Figure 11c).

We have shown that fractal dimension measures are a good instrument for measurement of urban morphology. This research is very helpful to provide a case to obtain a clear classification of the cities in different parts of the world, just as in the expectation in Tannier and Pumain [2]. However, the results obtained by fractal analysis are highly dependent on the generalization methods of the maps representing the urban surfaces that are used for the measurement of fractal dimension. So it is very important to accurately define “urban area” as a spatial object and carefully delineate the boundary of the urban; however, this is difficult and there is no universal consensus [41]. Almost every study employs its own criteria to delineate the study area. Even for the same study area, applying different methods, distinct conclusions could be drawn. Although we clearly defined the urban area, we did not base this definition on the jurisdiction of the cities but in the view of the real situation of China.

Urban sprawl intensity is used to normalize the urban sprawl area for the next comparison and analysis. Using spatial autocorrelation measure is an attempt to uncover the urban process over time in quantification, and it has proved to be practical and applicable. Especially it is very useful to detect the hot/cold spot transformation and then to reveal urban cluster pattern.

## 6. Conclusions

Urban research is very important and significant in any country, especially in a developing country such as China. One of the research means is to quantitatively measure the urban morphology and process. We also reveal the practicability of fractal dimension measures for homogeneity and compactness just as some literatures show. The difference is that the methods in this research are applied to analyze the evolution of urban cluster but not of inner urban area. Scaling behavior may help detect the change of the threshold range. The fractal dimension and its incidental scaling behavior may complement each other for homogeneity and compactness. Sprawl intensity is a good normalized index for different urban sprawls and also is a base to detect urban cluster pattern by spatial autocorrelation measure, which is very practical and applicable by Moran scatter map and hot/cold spot detection.

Since the implementation of economic reform and opening-up in China, the total urban area in Sunan zone has enlarged about 12-fold and the urban morphology is becoming more and more homogeneous and compact. The cities sprawl outwards their surroundings, especially along important transportation axes (e.g. railway, expressway); gradually, the cities stretching along the important transportation axes are attracted to each other and become much closer across space. Just as the process mentioned above, a big urban group, named by Suxichang urban group, is growing up in Sunan zone especially since 2000. The natural sprawl induced by an increase in urban population is much less than the industry-induced sprawl. Especially since 2000, rapid expansion of industrial development zones and/or economic and technical development zones is the main reason for the rapid urban sprawl. Sunan zone has a high level of spontaneous urbanization and we expect even greater urban sprawl in future years.

## Figures and Tables

**Table 1.** Global *I* of *SII* whose spatial weights are constructed based on contiguities from the nearest neighbors.

	**Threshold distance = 5,000 m**			**Threshold distance = 10,000 m**		

	**1984-1991**	**1991-2000**	**2000-2005**	**1984-1991**	**1991-2000**	**2000-2005**
***I*(*d*)**	1.620	0.200	0.456	0.707	0.167	0.222
***E*(*d*)**	-0.005	-0.005	-0.005	-0.005	-0.005	-0.005
***Z* Score**	15.317	1.878	4.165	18.517	4.363	5.666

**Table 2.** Global *G* of *SII* whose spatial weights are constructed based on contiguities from the nearest neighbors.

	**Threshold distance = 5000 m**			**Threshold distance = 10000 m**		

	**1984-1991**	**1991-2000**	**2000-2005**	**1984-1991**	**1991-2000**	**2000-2005**
***G*(*d*) (×10^-6^)**	9.234	2.165	1.517	19.57	7.300	5.689
***E*(*d*) (×10^-6^)**	1.034	1.034	1.034	4.454	4.454	4.454
***Z* Score**	17.053	3.475	2.441	19.084	5.229	3.668
